# Testicular Adrenal Rest Tumor Mimicking Leydig Cell Tumor in a Patient with Congenital Adrenal Hyperplasia

**Published:** 2014-04-01

**Authors:** Esra Karakuş, Müjdem Nur Azılı, Tuğrul Tiryaki

**Affiliations:** Department of Pathology, Ankara Children’s Hematology and Oncology Hospital, Turkey; Department of Pediatric Surgery, Ankara Children’s Hematology and Oncology Hospital, Turkey; Department of Pediatric Surgery, Ankara Children’s Hematology and Oncology Hospital, Turkey

**Dear Sir,**

A 7-year-old boy, known case of CAH, was referred to our hospital with bilateral testicular enlargement. On examination, hyperpigmentation of the scrotum was observed. In addition, ultrasound (US) of testicles revealed round-to-oval hypoechoic heterogeneous lesions [34x14 mm (right) and 19x7 mm (left)]. The patient underwent testicular sparing tumor enucleation. On macroscopic examination the nodules were well-circumscribed, encapsulated, firm, and yellow in color (Fig. 1). Microscopic evaluation revealed that the tumors were composed of polygonal cells with abundant eosinophilic cytoplasm and well-defined borders (Fig. 2). The tumor cells were separated by a fibrous stroma. There were no evidences of infiltrating margins, necrosis, nuclear atypia, or vascular invasion. There was also no evidence of Reinke’s crystals.

**Figure:**
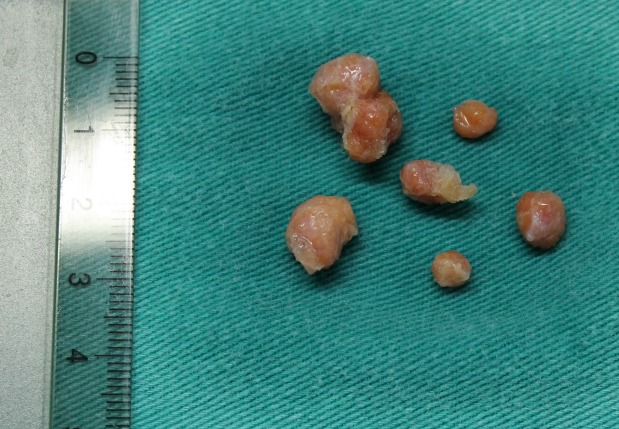
Figure 1: On gross examination the nodules appeared encapsulated, firm, and yellow in color

**Figure:**
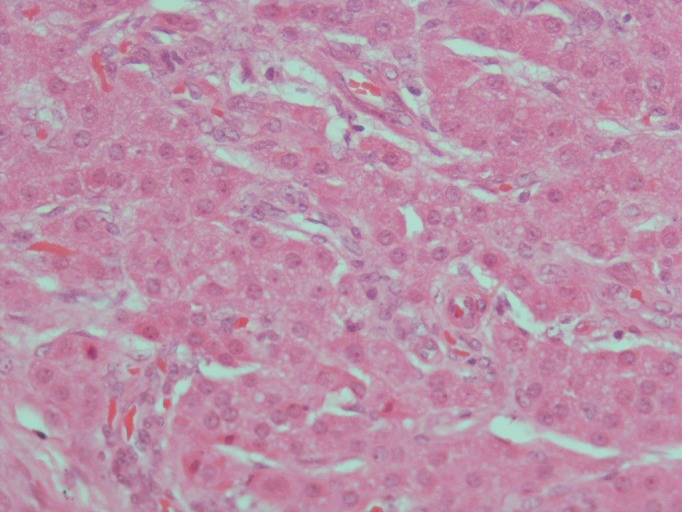
Figure 2: Tumor composed of polygonal cells with abundant eosinophilic cytoplasm and well-defined borders (X 400 H/E)

Congenital adrenal hyperplasia (CAH) is an autosomal recessive disorder characterized by enzyme defects in the steroidogenic pathways. Testicular adrenal rest tumors (TARTs) are may have serious consequences in patients with CAH. They probably develop from ectopic remnants of intratesticular adrenal tissue, which might be stimulated by the rise of adrenocorticotropic hormone (ACTH). Their histological differentiation from Leydig-cell tumors is extremely difficult.[1]

Testicular masses may be encountered in CAH patients which may lead to gonadal failure. These are bilateral lesions and are also called TART which do regresss after steroid therapy. Histopathologically they may be confused with Leydig cell tumors. TARTs are observed in young adults but are negative for Reinke crystals which are observed in Leydig cell tumors. Definitive diagnosis can be made by clinical, laboratory and endocrinological evaluations.[2,3]

## Footnotes

**Source of Support:** Nil

**Conflict of Interest:** None declared

